# Extraction of lichen bioactive compounds using volatile natural deep eutectic solvents and comparative analytical approaches

**DOI:** 10.1038/s41598-025-08069-0

**Published:** 2025-07-02

**Authors:** S. Dresler, I. Baczewska, O. Mykhailenko, Ch. Zidorn, I. Sowa, M. Wójciak, M. Feldo, H. Wójciak, A. Hanaka, M. Strzemski

**Affiliations:** 1 Department of Analytical Chemistry, Medical University of Lublin, Chodźki 4a, 20-093 Lublin, Poland; 2 Department of Plant Physiology and Biophysics, Institute of Biological Sciences, Maria Curie-Skłodowska University, Akademicka 19, 20-033 Lublin, Poland; 3 Department of Pharmaceutical Chemistry, National University of Pharmacy, 61168 Kharkiv, Ukraine; 4 School of Pharmacy, University College London, 29-39 Brunswick Square, London, WC1N 1AX UK; 5 Department of Pharmaceutical Biology, Kiel University, 24118 Kiel, Germany; 6 Division of Pharmaceutical Biotechnology, Department of Pharmaceutical, Biology and Biotechnology, Wroclaw Medical University, Borowska 211, 50-556 Wrocław, Poland; 7 Department of Vascular Surgery, Medical University of Lublin, Staszica 11, 20-081 Lublin, Poland; 8 Institute of Biological Sciences, Maria Curie-Skłodowska University, Akademicka 19, 20-033 Lublin, Poland

**Keywords:** *Hypogymnia physodes*, Accelerated solvent extraction, Lichens, VNADES, Bioactive compounds, Green extraction, Plant sciences, Chemistry

## Abstract

Lichens are a source of bioactive secondary metabolites with different pharmacological activities. However, these metabolites are challenging regarding their extraction. The presented study aims to optimise the extraction methods of key compounds (depsidones and depsides) from *Hypogymnia physodes* (L.) Nyl. and additional species of lichens using traditional and advanced extraction methods. The comparative analysis included the evaluation of accelerated solvent extraction (ASE), maceration method of extraction, and using of volatile natural deep eutectic solvents (VNADES), as well as the type of solvent, ratio, duration and temperature of extraction on the yield of the main lichen metabolites (physodalic and physodic acids, 3-hydroxyphysodic acid, atranorin, and chloroatranorin). The combination of ASE with acetone was found to be the most selective method for lichen acid extraction, reducing both solvent consumption and processing time. As a green extraction approach, the use of VNADES was evaluated for yield. The following extraction parameters using VNADES were developed to maximise metabolite extraction: menthol/camphor ratio (1.5:1), liquid/solid ratio (100:1, volume/mass), temperature of extraction (40 °C), time of extraction (30 min). A comparison of different extraction drying methods showed the effectiveness of rotary evaporation as a cost-effective and rapid alternative to lyophilisation, preserving bioactive compounds from degradation.

**Supplementary Information:**

The online version contains supplementary material available at 10.1038/s41598-025-08069-0.

## Introduction

Recently, there has been a strong interest in products obtained from renewable raw materials as an alternative to industrial organic synthesis products^1^. This is due to a number of reasons, one of which is the impossibility of synthesising many complex natural compounds. Secondary metabolites obtained from natural sources often display high biological activity. The search for antimicrobial natural products is especially relevant, given the growing problem of antibiotic resistance^2^. Epiphytic lichens are sources of lichen acids, flavonoids, and other low molecular weight phenols.

Lichens are specialised group of symbiotic organisms whose body consists of two (or more) partners: a fungus (or mycobiont) and an alga (or photobiont)^3^. This combination provides lichens with water and various chemical elements from the environment, which accumulate in the thalli of these lower plants, giving them a unique biochemical composition. Secondary metabolites are the most important lichen components, which are produced by the mycobiont in symbiosis with the phycobiont and are located on the walls of the hyphae. The most well-known secondary metabolites are atranorin, fumarprotocetraric acid, gyrophoric acid, salazinic acid, and usnic acid^4,5^. Due to the presence of these metabolites, liches exhibit antibacterial, antiviral, anti-inflammatory, analgesic, and antipyretic effects^6^ and have long been widely used in traditional medicine for the treatment of wounds, skin diseases, breathing and digestive issues^7^. It should be noted that the use of lichens as raw materials for pharmaceutical developments can be considered as an additional use of non-timber forest products.

One of the prominent representatives is *Hypogymnia physodes* (L.) Nyl. (Parmeliaceae family). This polymorphic foliose lichen, also known as Monaca’s hood, is widespread in temperate and boreal forests of the Northern Hemisphere^8^ especially in northern Asia, North America, and Europe. *H. physodes* is growing mainly on the trunks and branches of conifers, birch, and other types of deciduous trees. It contains various active compounds such as physodic acid, physodalic acid, 3-hydroxyphysodic acid, 2′-*O*-methylphysodic acid, protocetraric acid, chloroatranorin, and atranorin^9^. The mentioned depsidones and depsides and lichen extracts in general exhibit a wide range of pharmacological properties, including antimicrobial, antioxidant, anti-inflammatory, and anticancer activities^10,11^. Physodic and usnic acids, as well as atranorin from *H. physodes* (of Serbian origin) extract showed very strong antimicrobial activity against to *Bacillus mycoides*,* Bacillus subtilis*,* Klebsiella pneumonia*, and *Staphylococcus aureus*^11^. A similar pattern of antimicrobial activity was established for acetone (Ac) extract of *H. physodes* (Turkey origin) against *Escherichia coli*,* Bacillus subtilis*,* Bacillus megaterium* due to the higher content of usnic acid (1.05% of dry weight in the test sample)^12^.

Another compound commonly found in lichens, chloroatranorin, also showed significant antimicrobial effects against pathogenic bacteria such as *Aeromonas hydrophila*, *B. cereus*,* B. subtillus*, *S. aureus*^13^. At the same time, atranorin isolated from *Cladonia foliacea*^14^ had the low antimicrobial activity against nine different bacterial microorganisms, but according to Stojanowic et al.^15^ atranorin from *H. physodes* was active against *C. albicans*. This suggests that the compound isolated from different lichen species may exhibit activity in different ways and it is important to find the optimal method for its extraction and isolation. Physodalic acid from the same lichen also possesses antioxidant and antimicrobial activity. Atranorin also exhibits antioxidant^16^ antiviral, antiprotozoal, and phytoprotective activity^17^ and the presence of this compound in different lichen species^18^ emphasises the importance of phytopharmaceutical developments with this type of raw material.

Cytotoxic activity of Ac extract from *H. physodes* and its major component, i.e., physodic acid was also established. It demonstrated significant effect against glioblastoma cell lines A-172, T98G, and U-138 MG, and inhibited the activity of tyrosinase, an enzyme associated with neurodegenerative diseases^19^ which is important for the development of the medicines for the prevention and treatment of Alzheimer’s and Parkinson’s disease as well as brain tumors. In another study^11^ physodic acid and atranorine showed significant cytotoxic activity against human melanoma and human colon carcinoma cell lines.

Evernic acid isolated from *Evernia prunastri* lichen also shown neuroprotective anti-inflammatory effects^20^ and extracts from this lichen species were strongly active against *S. aureus*^21^. Other studies also describe the antioxidant, antimicrobial and antiproliferative properties of extracts of the lichens *Parmelia sulcata*,* Flavoparmelia caperata*,* E. prunastri*,* H. physodes*, and *Cladonia foliacea* due to the higher content of phenolic compounds^10,22,23^ and activity of individual compounds isolated from them^24^.

The rich composition of secondary metabolites provide potential for *H. physodes* and various *Hypogymnia* species as a source of pharmacologically active compounds for further drug development or functional food applications^21^. However, despite extensive studies on lichens, challenges remain in isolating individual compounds and developing technological approaches to obtain the total content of compounds from lichens. Notably, their complex structure – composed of fungal and algal/cyanobacterial components enclosed in a robust protective matrix – presents a significant barrier^3^. Additionally, the low solubility of lichen metabolites further complicates their extraction^25^. Moreover, the described studies mainly concern the isolation of the most abundant and well-studied phenolic metabolite of lichens – usnic acid, while other compounds have not been investigated as extensively.

The described technological approaches, including Soxhlet extraction^26^ supercritical fluid extraction^27^ and microwave-assisted extraction^28^ are promising, but need to be improved for greater efficacy. Moreover, they may cause degradation of sensitive metabolites^29^. Among other factors, the use of aggressive organic solvents such as acetone (Ac) benzene, hexane, ethanol, petroleum ether, chloroform or their mixtures can also destroy the structure of lichen acids if not properly combined and proportioned. Therefore, for lichen metabolites, it is crucial to apply an optimal approach that ensures not only the preservation of sensitive components but also their complete extraction, as well as the reproducibility and scalability of process parameters.

Our previous studies have shown that volatile natural deep eutectic solvents (VNADES) represent an optimal modern approach for extracting compounds from lichens. Using the example of usnic acid extraction from *Cladonia uncialis* (L.) Weber ex F.H. Wigg., the efficiency of a mixture of thymol and camphor as natural deep eutectic solvents (NADES) was demonstrated. The results indicated an optimised extraction procedure, enabling the recovery of approximately 13% more total usnic acid in a single extraction compared to classical Ac-based extraction^30^. Another example of the use of NADES solutions based on proline or betaine and lactic acid for the extraction of metabolites from the thalome of *H. physodes* should also be mentioned^4^ as it allowed the efficient extraction of three depsidones (physodic acid, physodalic acid, 3-hydroxyphysodic acid) and one depside (atranorin), after developing optimal solvent mixture ratios.

In support of advanced NADES approaches, it is important to highlight their environmental friendliness, efficiency, biocompatibility, rapid application in the extraction of bioactive components, minimal by-product formation during extraction. They also reduction of dependence on harmful organic solvents, and other advantages, Furthermore, all of these benefits align with the principles of green chemistry^31^. VNADES are composed of volatile organic compounds (e.g., menthol, thymol, and camphor) that can easily evaporate under certain conditions, simplifying the extraction process and minimising solvent residues. Additionally, VNADES have demonstrated excellent efficiency in extracting sensitive metabolites, such as polyphenols, flavonoids^32^, alkaloids^33^ and lichen acids^4^ without degradation.

Therefore, it is important to compare traditional and modern extraction methods with VNADES to identify effective and sustainable approaches for extracting key lichen compounds. This study presents an effective alternative to conventional solvents for the extraction of lichen metabolites.

## Results and discussion

Considering the importance of bioactive metabolites of lichens, the current study aimed to optimise the extraction conditions of the main marker compounds on the example of *H. physodes*.

### Thermolability of *H. physodes* metabolites

**Fig. 1:**
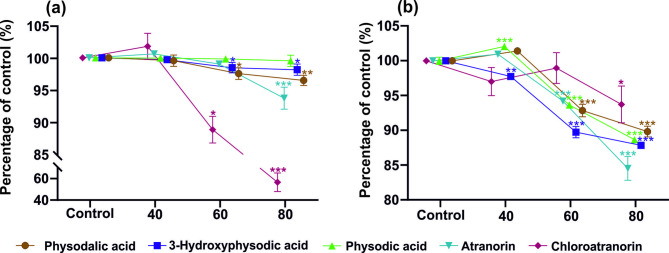
Thermostability of physodalic acid, 3-hydroxyphysodic acid, physodic acid, atranorin, and chloroatranorin at three temperatures (40 °C, 60 °C, and 80 °C), with the control sample stored at room temperature. (**a**) A mixture of lichen compounds dissolved in DMSO was subjected to a 15-min incubation in an incubator at various temperatures. (**b**) A mixture of lichen compounds dissolved in acetone was exposed to temperature treatment using an accelerated solvent extraction (ASE) system. Data are mean ± SD (*n* = 3), *p < 0.05, **p < 0.01, ***p < 0.001 compared to the control.

Temperature is one of the most significant factors affecting the efficiency of the extraction of active compounds from plant material^34^. However, high temperature can increase the extraction level of metabolites by changing the polarity and viscosity of the solvent or by increasing the diffusion and solubility of the extracted analyte. At the same time, increasing the temperature above the critical value can lead to the degradation of metabolites^35^. In order to determine the potential degradation of lichen metabolites during extraction at higher temperatures, two tests were conducted to determine the effect of temperature on the stability of compounds in two different systems (Fig. 1).

The first test, using dimethyl sulfoxide (DMSO) extract solutions subjected to different temperatures (Fig. 1a), showed that the most thermolabile compound was chloroatranorin, whose content decreased by more than 11% and 44% in solutions treated at 60 °C and 80 °C, respectively. In addition, there were slight but significant decreases in 3-hydroxyphysodic and physodalic acids content at 60 °C and in atranorin, 3-hydroxyphysodic, physodalic and physodic acids at 80 °C.

In the second test using Ac and the ASE technique, the lichen acids were subjected to both different temperatures and high pressure. At 80 °C, a significant reduction in the content of all the compounds was observed (Fig. 1b). Similarly, the temperature of 60 °C significantly decreased the content of lichen acids, with the exception of chloroatranorin. On the other hand, a temperature of 40 °C slightly but significantly decreased the content of 3-hydroxyphysodic acid and increased the concentration of physodic acid. It can be assumed that the temperature of 40 °C combined with high pressure initiated the process of dehydroxylation of the phenolic ring of 3-hydroxyphysodic acid, leading to an increase in the pool of physodic acid at the expense of the former (Fig. 1b). Therefore, to reduce the possibility of degradation of lichen acids due to high temperature, the extraction process, regardless of the technique tested, was performed at a temperature not exceeding 40 °C.

### Comparison of extraction methods

**Fig. 2:**
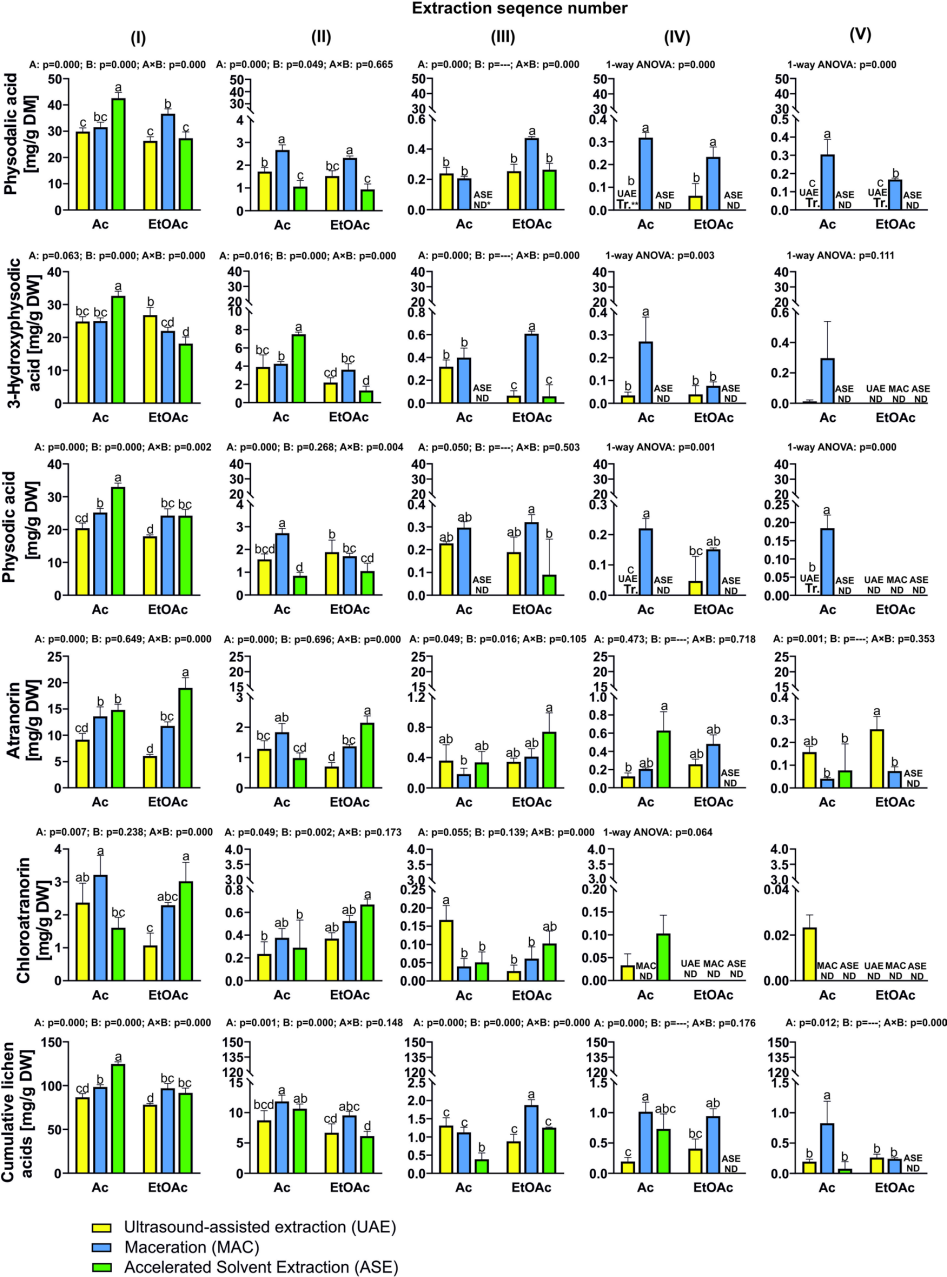
Effect of solvent type (acetone—Ac; ethyl acetate—EtOAc) and extraction method (ultrasound-assisted extraction—UAE; maceration—MAC; accelerated solvent extraction—ASE) on the extraction of physodalic acid, 3-hydroxyphysodic acid, physodic acid, atranorin, chloroatranorin, and the total content of lichen acids (mg/g DW) from *H. physodes* during successive extraction steps using fresh solvent. *Values below the limit of detection (LOD); **trace amounts below 1 ppm. Columns marked with different lowercase letters represent significant differences based on Tukey’s post-hoc test (*p* < 0.05). Two-way ANOVA results: p-values indicate the effects of factors: A (extraction method), B (solvent type), and A × B (interaction between method and solvent). Data are presented as means (*n* = 3) ± standard deviation (SD).

One of the key factors affecting the efficiency of secondary metabolite extraction from lichens is the choice of extraction method. Traditional approaches using maceration (MAC), percolation or Soxhlet extraction are inefficient and time-consuming. In addition, conventional extraction methods generally require significant amounts of solvent^36^. However, advanced extraction techniques such as accelerated solvent extraction (ASE) and ultrasound-assisted extraction (UAE) are designed to accelerate the extraction process through the application of heat, pressure, or ultrasonic waves. These modern extraction techniques use less solvent and energy than traditional methods, while achieving higher extraction efficiencies, and they can be considered as techniques in line with green chemistry approaches^36,37^. The efficiency of three extraction methods (MAC, ASE and UAE) using two types of solvent (Ac and ethyl acetate (EtOAc)) was tested (Fig. 2, Supplementary Fig. 1). It was found that for the three main depsidones – physodalic acid, 3-hydroxyphysodic acid, and physodic acid – and for the cumulative of lichen acids, the highest first stage extraction efficiencies were achieved with Ac and ASE (Fig. 2). For these three depsidones, the efficiency of Ac extraction in the first step with UAE and MAC ranged from 62 to 76% of the extraction level achieved with ASE and Ac. Additionally, it was pointed out that the association of ASE and Ac achieved a depletion of the depsidones already in the second extraction step.

The combination of ASE with Ac was particularly effective compared to MAC, where the 3 main depsidones were still extracted in the fifth extraction step with Ac and, in the case of physodalic acid, also with EtOAc. Acetone and EtOAc have been reported in the literature as some of the most effective extractants of lichen acids^4^. Kulinowska et al.^30^ confirmed that both solvents provide a good medium for the dissolution of usnic acid, with values of 637 mg/100 mL for Ac and 657 mg/100 mL for EtOAc. The efficiency of EtOAc in the extraction of depsidones was comparable to that of Ac in ultrasound-assisted extraction. This result was somewhat surprising, as previous studies had found EtOAc to be more efficient than Ac in a one-step UAE extraction^4^. However, in the case of MAC, despite the fact that the first extraction steps did not differentiate between Ac and EtOAc, the EtOAc extraction led to a faster depletion of 3-hydroxyphysodic acid and physodic acid by the 4th extraction step. On the other hand, EtOAc was significantly less efficient than Ac in the extraction of the three major lichen acids of *H. physodes* by the ASE method. The efficiency of EtOAc, assessed by the amount of metabolite extracted using ASE, was 64.1%, 55.5%, and 73.3% of the amount of physodalic acid, 3-hydroxyphysodalic acid and physodic acid extracted with Ac, respectively. In contrast to the depsidones, the extraction of atranorin and chloroatranorin with EtOAc using ASE was significantly more effective than with Ac, and the depletion of these components was already observed in the third extraction step with EtOAc. In contrast, the type of solvent did not affect the extraction of atranorin in the first extraction step using UAE and MAC, nor that of chloroatranorin using MAC. However, UAE with EtOAc extracted significantly less chloroatranorin than Ac in the first extraction step.

**Fig. 3:**
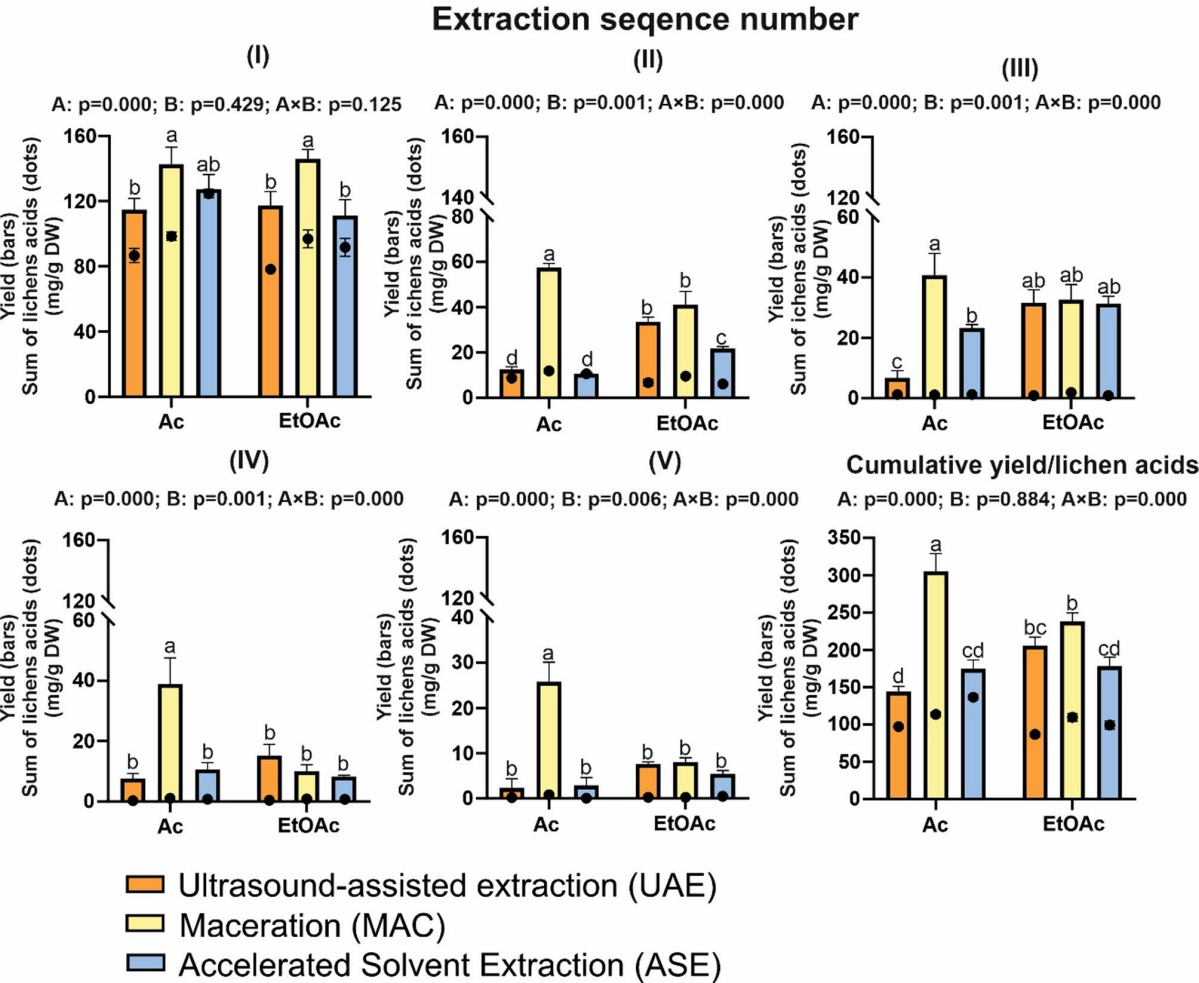
Effect of extraction method: ultrasound-assisted extraction (UAE), maceration (MAC), and accelerated solvent extraction (ASE), and solvent type: acetone, ethyl acetate on extraction yield (bars) and total lichen acid content (dots) in successive stages of the extraction sequence (I-V) and for the total amount collected from all extraction steps. Data are mean ± SD (*n* = 3). Different letters indicate significant differences between the samples, calculated based on a 2-way ANOVA and post-hoc Tukey analysis Two-way ANOVA results: p-values indicate the effects of factors: A (extraction method), B (solvent type), and AxB (interaction between method and solvent). Data are presented as means (*n* = 3) ± standard deviation (SD).

It is noted that one of the most important properties of solvents, which determines their usefulness in extracting metabolites, is their polarity^38^. Ethyl acetate has a slightly lower polarity (Log*P* 0.7) than Ac (Log*P* − 0.1). This means that compounds with a higher lipophilicity will dissolve better in this solvent^39^. It appears that differences in these solvent properties are one of the main sources of variability in the usefulness of solvents in the extraction of metabolites from plants. However, comparing the results obtained with previous studies, it should be noted that in comparison to other common solvents, both Ac and EtOAc exhibited high efficiency in extracting specialised lichen substances^4^. Nevertheless, their performance may vary when combined with various modern extraction techniques. In addition to the efficiency of selective extraction of selected metabolites, the overall extraction yield was also evaluated (Fig. 3). It was found that in the first extraction step, only the extraction method was significant in the total extraction yield. In general, the 24-h MAC resulted in a higher overall yield. In contrast, the subsequent exhaustive extraction steps indicate a significant interaction effect between the type of extractant and the extraction method.

Overall, the long-term MAC with Ac in successive extraction steps was much more effective in achieving high yields than other methods. In the case of EtOAc, the extraction level (except for the significantly lower extraction of ASE in step 2) was at a similar level between the methods. Finally, the combination of MAC and Ac resulted in an average 2-fold higher cumulative yield compared to UAE and ASE. It is significant that MAC, the most traditional method and considered the least effective, gave the highest yield^40^. On the other hand, the combination of MAC + Ac was unusually the least selective for specific lichen acids, despite the high yield (Fig. 3). Undoubtedly, the ASE + Ac combination had the highest selective extraction of lichen acids, with this method achieving a yield in the first extraction (Fig. 3.I) of 95% by weight lichen acids of *H. physodes*. The cumulative effect of the extraction also demonstrated the high selectivity of this method, with more than 78% of the total extraction yield consisting of specific lichen acids. Relatively high selectivity was also shown by extraction in the combination of UAE + Ac, but this system was less effective.

Nevertheless, the methods using EtOAc showed low selectivity, with the lowest selectivity expressed by the ratio of total yield to extract specific lichen metabolites was observed for the extraction of MAC + Ac. This low selectivity of the MAC + Ac combination method was due to the very high overall yield for this method (over 300 mg/g). It can be assumed that the longer maceration time (24 h) allowed the extraction of a wider range of both lipophilic and hydrophilic compounds. Although the traditional MAC method is considered to be a less efficient extraction method, prolonged MAC has also previously been shown as a method that achieves a better overall extraction yield of phenolic compounds compared to the UEA method^41^. However, our results indicate that for the selective extraction of lichen acids, the best approach is the ASE + Ac combination, which ensures not only high selectivity but also saves time and reduces solvent consumption.

### Optimisation of extraction using hydrophobic VNADES

The use of natural deep eutectic solvents (NADES) for of plant metabolites extraction has been seen in recent years as an alternative extraction method that reduces the use of often organic toxic solvents, in line with the philosophy of green chemistry^42^. However, NADES also have some limitations primarily due to their high viscosity level, which hinders effective mixing the solvent with the plant matrix. In addition, these mixtures have a very low volatility which precludes the possibility of solvent evaporation^33^. This problem can be partially solved by using volatile NADES components such as menthol and camphor, which allow the evaporation of extractants. In the present study, four key parameters of extraction were optimised using menthol-camphor VNADES by ultrasonic assisted extraction (UAE) method. Although previously described results of high extraction efficacy of ASE combined with Ac has been showed (Fig. 2), the obtained results of extraction in combination of ASE and VNADES was low effective (data not shown) due to high viscosity of VNADES and technical problems with dosing and flow of solvents by ASE equipment. Menthol/camphor ratio (*X*_*1*_), liquid/solid ratio (*X*_*2*_), temperature (*X*_*3*_) and extraction time (*X*_*4*_) were investigated as the main variables affecting the efficiency of the extraction process. These factors are identified as key parameters in optimising the extraction of biologically active compounds from plants^43^. The optimal menthol/camphor ratio of 1.5 to 9 was determined through screening mixture tests (Suppl. Figure 2). Due to the observed thermal sensitivity of the lichen compounds, the maximum extraction temperature was set at 40 °C. The range of the liquid/solid ratio (L/S) was established based on experimental evaluation of the raw material’s wetting potential by the liquid, ensuring effective extraction.

On the basis of the extractions carried out according to the established experimental design, polynomial regression models were developed for each of the individual compounds as well as for their cumulative values (Table 1). The obtained models were optimised by removing terms with a high level of insignificance (*p* > 0.1). The final models demonstrated high statistical significance and showed no significant lack of fit. The developed models had a coefficient of determination (*R²*) ranging from 0.58 to 0.69, while predicted *R*^2^ between 0.42 and 0.60 which is in reasonable agreement with the adjusted *R²*, as indicated by a difference of less than 0.2^44^. Additionally, all models exhibited a high signal-to-noise ratio, as evidenced by an Adeq Precision value greater than 4.

**Fig. 4:**
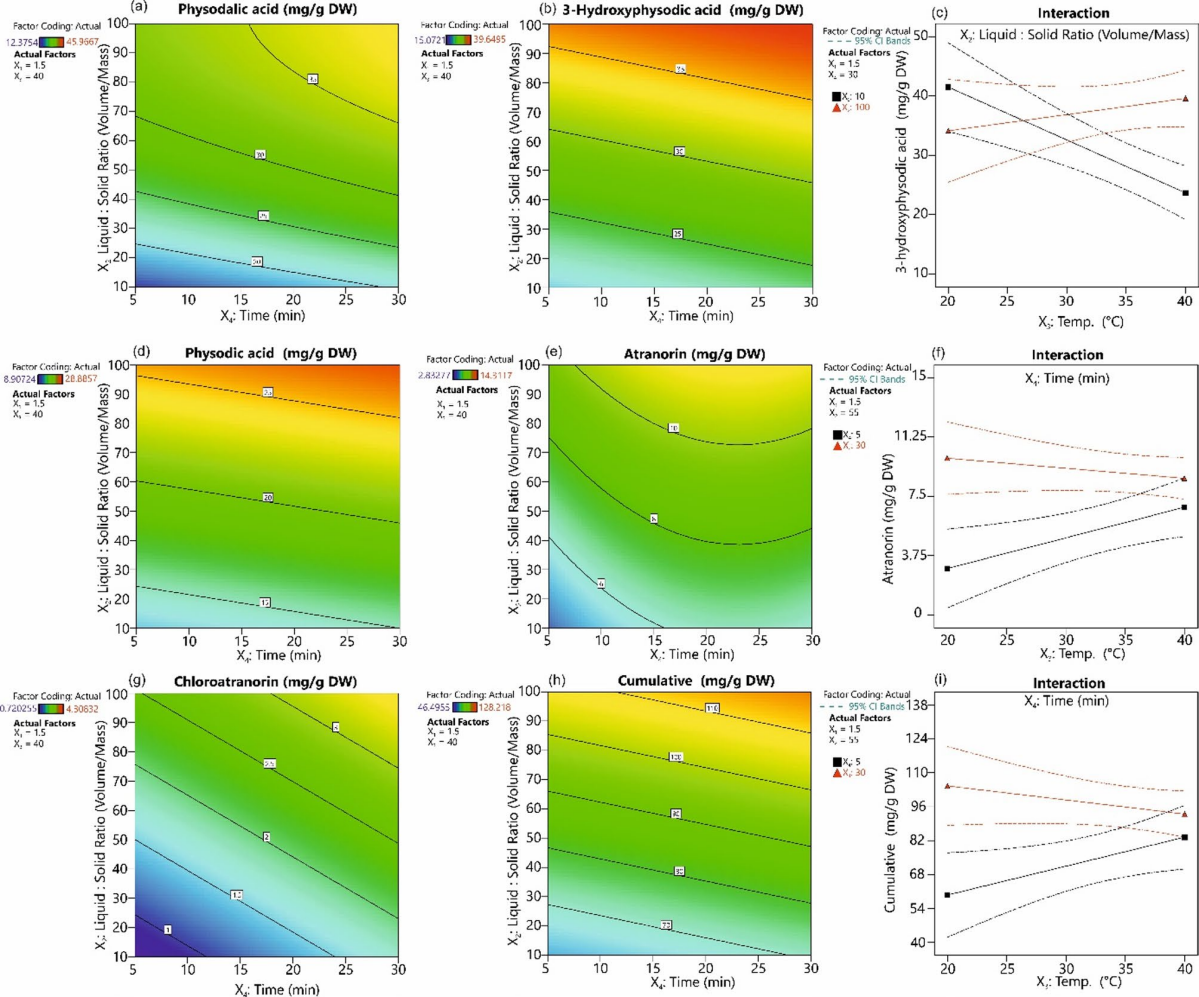
Response surface plots and interaction plots for the influence of factors on the extraction efficiency of specialised metabolites of *H. physodes* using volatile natural deep eutectic solvents (VNADES). (**a**) The effect of liquid/solid ration (*X*_*2*_) and time (*X*_*4*_) on physodalic acid extraction yield. (**b**) The effect of liquid/solid ration (*X*_*2*_) and time (*X*_*4*_) on 3-hydroxyphysodic acid extraction yield; (**c**) the interaction effects of liquid/solid ration (*X*_*2*_) and temperature on 3-hydroxyphysodic acid extraction yield; (**d**) the effect of liquid/solid ration (*X*_*2*_) and time (*X*_*4*_) on physodic acid extraction yield; (**e**) the effect of liquid/solid ration (*X*_*2*_) and time (*X*_*4*_) on atranorin extraction yield; (**f**) the interaction effects of time (*X*_*4*_) and temperature (*X*_*3*_) on 3-hydroxyphysodic acid extraction yield; (**g**) the effect of liquid/solid ration (*X*_*2*_) and time (*X*_*4*_) on chloroatranorin extraction yield; (**h**) the effect of liquid/solid ration (*X*_*2*_) and time (*X*_*4*_) on cumulative lichen acids extraction yield; (**i**) the interaction effects of time (*X*_*4*_) and temperature (*X*_*3*_) on cumulative lichen acids extraction yield.

Surprisingly, the tested range of the menthol-to-camphor molar ratio did not significantly affect the extraction efficiency of any individual metabolite. Although it is generally suggested that the ratio of individual components in VNADES can influence the solvent’s viscosity^30^, this effect was limited and insignificant in the present study. On the other hand, the L/S ratio proved to be a significant factor, with its increase leading to higher extraction efficiency (Table 1) (Fig. 4a,b,d,e,g,h). This phenomenon has been confirmed in previous studies^4^ and is explained by Fick’s first law^45^. At a higher L/S ratio, equilibrium between the phases is established more slowly, allowing the concentration gradient to be maintained for a longer period. This sustains a stronger driving force for diffusion, enhancing mass transfer. Additionally, a high L/S ratio improves solvent penetration into the solid matrix and reduces the impact of high solvent viscosity, which could otherwise hinder diffusion^46^. Furthermore, a higher L/S ratio minimizes the risk of solvent saturation, thereby reducing solute reabsorption and precipitation, ultimately improving extraction efficiency. A significant interaction between L/S ratio and temperature was observed only in the case of 3-hydroxyphysodalic acid. The extraction efficiency of this compound was higher at a low L/S ratio and low temperature compared to high temperature (Fig. 4c). This phenomenon may be due to the higher thermal sensitivity of 3-hydroxyphysodalic acid at 40 °C, compared to other compounds (Fig. 1b). The interactive effect of these two factors indicates a complex balance between solubility, diffusion dynamics, and thermal stability of 3-hydroxyphysodalic acid. We assume that at a low L/S ratio, the higher local concentration of the metabolite in the solvent makes it more susceptible to thermal degradation. Conversely, a high L/S ratio reduces the local concentration of the metabolite, providing protection against thermal degradation. In contrast to previous findings, where extraction time (10–30 min) had no significant effect on the extraction efficiency of *H. physodes* metabolites using NADES based on proline/betaine and lactic acid^4^, our study indicates that extending the extraction time significantly enhances extraction efficiency (Fig. 4a,b,d,e,g,h). Considering previous research on VNADES and the extraction of usnic acid from *Cladonia unicialis* (L.), where prolonged extraction improved efficiency^30^ as well as the results presented here, it can be concluded that VNADES requires a longer time to reach equilibrium of concentrations compared to the non-volatile NADES. An interesting phenomenon is the observed interaction between temperature and extraction time (Fig. 4f,i). It can be assumed that, in a short extraction time, higher temperatures enhance diffusion processes, thereby increasing extraction efficiency. On the other hand, a prolonged extraction time combined with high temperature may lead to the partial degradation of the extracted compounds. This interaction suggests that the optimal extraction conditions depend on balancing temperature and time to maximise diffusion while minimising thermal degradation.

**Table 1:** Fitting statistics, analysis of variance and regression coefficients of the models constructed for each specialised metabolite of *H. physodes*. Variable coded: *X*_*1*_: menthol/camphor ratio; *X*_*2*_: liquid/solid ratio (volume/mass); *X*_*3*_: temperature of extraction (°C); *X*_*4*_: time of extraction (min).

Physodalic acid			3-Hydroxyphysodic acid		
R^2^	0.6773		R^2^	0.5825	
Adjusted R^2^	0.6325		Adjusted R^2^	0.5109	
Predicted R^2^	0.5640		Predicted R^2^	0.4185	
Adeq Precision	12.6736		Adeq precision	10.7845	

Term	Coefficient estimate	*p*-value	Term	Coefficient estimate	*p*-value
Intercept	28.72		Intercept	37.05	
$\:{X}_{1}$	− 0.0529	0.9626	$\:{X}_{1}$	− 1.53	0.1739
$\:{X}_{2}$	8.86	< 0.0001	$\:{X}_{2}$	2.14	0.1006
$\:{X}_{3}$	1.61	0.1814	$\:{X}_{3}$	− 0.0489	0.9667
$\:{X}_{4}$	2.68	0.0188	$\:{X}_{4}$	4.66	0.0005
$\:{\text{X}}_{2}^{2}$	− 3.87	0.0270	$\:{X}_{2}{X}_{3}$	5.82	0.0009
			$\:{X}_{3}{X}_{4}$	− 3.04	0.0597
Model		< 0.0001	Model		< 0.0001
Lack of fit		0.3149	Lack of fit		0.4545

Physodic acid			Atranorin		
R^2^	0.6899		R^2^	0.6722	
Adjusted R^2^	0.6468		Adjusted R^2^	0.616	
Predicted R^2^	0.5984		Predicted R^2^	0.5487	
Adeq Precision	13.9396		Adeq Precision	13.3099	

Term	Coefficient estimate	*p*-value	Term	Coefficient estimate	*p*-value
Intercept	18.77		Intercept	8.37	
$\:{X}_{1}$	− 0.1615	0.8375	$\:{X}_{1}$	0.2526	0.5214
$\:{X}_{2}$	6.25	< 0.0001	$\:{X}_{2}$	2.64	< 0.0001
$\:{X}_{3}$	1.33	0.1143	$\:{X}_{3}$	0.6491	0.1299
$\:{X}_{4}$	3.16	0.0008	$\:{X}_{4}$	2.20	< 0.0001
$\:{X}_{3}{X}_{4}$	− 2.16	0.0546	$\:{X}_{3}{X}_{4}$	− 1.29	0.0234
			$\:{\text{X}}_{3}^{2}$	− 1.04	0.0797
Model		< 0.0001	Model		< 0.0001
Lack of fit		0.537	Lack of fit		0.6752

Chloroatranorin			Total lichen metabolites		
R^2^	0.6424		R^2^	0.6908	
Adjusted R^2^	0.6037		Adjusted R^2^	0.6478	
Predicted R^2^	0.5544		Predicted R^2^	0.5951	
Adeq Precision	15.7748		Adeq Precision	14.5509	

Term	Coefficient estimate	p-value	Term	Coefficient estimate	p-value
Intercept	2.11		Intercept	84.33	
$\:{X}_{1}$	0.1696	0.1786	$\:{X}_{1}$	− 1.72	0.5634
$\:{X}_{2}$	0.8749	< 0.0001	$\:{X}_{2}$	23.22	< 0.0001
$\:{X}_{3}$	0.1674	0.1983	$\:{X}_{3}$	3.12	0.3221
$\:{X}_{4}$	0.5128	0.0001	$\:{X}_{4}$	13.94	0.0001
			$\:{X}_{3}{X}_{4}$	− 9.06	0.0343
Model		< 0.0001	Model		< 0.0001
Lack of fit		0.4449	Lack of fit		0.5643

### Response prediction and model confirmation

Based on the developed model, the optimal conditions for the extraction of lichen acids using VNADES were determined as follows: menthol/camphor ratio – 1.5:1; L/S ratio – 100:1; extraction temperature – 40 °C; extraction time – 30 min. These conditions maximised the extraction of lichen acids from *H. physodes*. To confirm the model, an extract was prepared under these optimised conditions, and the amount of lichen specialised compounds was determined and compared with the predicted values (Table 2). The predicted and experimental values for the sum of lichen acids were found to be in good agreement with relative deviations (RDs) below 4%. On the other hand, individual compounds showed a somewhat poorer correlation of predictive values to those obtained experimentally, with RDs ranging from 8 to 18%, although these values remained within the 95% prediction interval (PI).

**Table 2:** Predicted values and experimental mean values (mg/g DW) at the optimal extraction conditions for *X*_*1*_: menthol/camphor ratio (1.5:1); *X*_*2*_: liquid/solid ratio (100:1, volume/mass); *X*_*3*_: temperature of extraction (40 °C); *X*_*4*_: time of extraction (30 min).

Response variables	Predicted mean	Observed mean value	Std dev	RD (%)	95% PI low	95% PI high
Physodalic acid (mg/g DW)	38.05	32.28	5.163	17.875	30.570	45.529
3-Hydroxyphysodic acid (mg/g DW)	39.60	35.02	5.049	13.078	31.990	47.200
Physodic acid (mg/g DW)	27.52	29.96	3.575	− 8.144	22.361	32.670
Atranorin (mg/g DW)	11.28	12.48	1.783	− 9.615	8.689	13.871
Chloroatranorin (mg/g DW)	3.50	3.09	0.572	13.269	2.683	4.3105
Cumulative lichen acid (mg/g DW)	117.29	112.82	13.517	3.962	97.799	136.779

### Optimised extraction method for metabolite analysis in different lichen species

VNADES with an optimal molar ratio of menthol and camphor (1.5:1) was used to extract metabolites from seven lichen species and the extraction efficiency of these compounds was compared with that obtained using Ac. Nine compounds were found in the analysed material (Table 3), with atranorin being the most abundant. This compound was detected in all lichens except *C. uncialis*, confirming the considerable its prevalence in various lichen species, especially those listed in Table 3^47–49^. It and may also indicate significant variation in the chemical composition of *C. uncialis*, in which atranorin was previously identified^21^.

Interestingly, the use of VNADES and Ac gave different yields of atranorin extraction depending on the lichen extracted. It is not possible to determine which solvent is a better extractant of this compound overall. In the case of *H. physodes* and *P. sulcata*, no significant differences were found between the atranorin extraction efficiencies with VNADES and Ac. However, Ac proved to be a more effective extractant for *P. furfuracea*, *E. prunastri*, and especially *P. adscendens*. Conversely, the use of VNADES resulted in better atranorin extraction from *X. parietina* compared to Ac. It should be noted that the VNADES used was an efficient extractant of atranorin from lichens with relatively low atranorin content, while Ac proved more effective in extracting matrices containing significant amounts of this compound.

Similarly, the extraction efficiency of chloroatranorin was at the same level from the raw material containing a small amount of this compound (*P. furfuracea*), whereas in the case of *H. physodes* and *E. prunastri*, which contained relatively much chloroatranorin, Ac turned out to be a more efficient extractant. Evernic acid was extracted more efficiently by VNADES from *P. furfuracea* (which contains low level of this compound), while Ac was more effective extractant for *E. prunastri* (which is rich in evernic acid).

The remaining compounds were determined in a few of the lichen species, so it was not possible to assess the relationship between extraction efficiency and content in the raw material. However, no significant differences were found in the extraction efficiency of physodalic acid from *H. physodes*, usnic acid from *C. uncialis*, physcion from *X. parietina* and salazinic acid from *P. sulcata* using VNADES and Ac. However, Ac was a better extractant for 3-hydroxyphysodic acid and physodic acid from *H. physodes*. These observations support the principle that there are no universal solvents that can be used for the efficient extraction of a wide range of compounds, however the proposed VNADES may provide an alternative to Ac for the extraction of lichen metabolites. The data presented here suggest that the extraction efficiency of these metabolites with VNADES can be improved in the future by using appropriate extraction techniques. The less efficient extraction of some compounds with VNADES may be due to the matrix structure or slower mass transfer in a more viscous solvent. On the other hand, the efficiency does not seem to be affected by the limited solubility of e.g., atranorin, since the relationship between the content of this compound in extracts made with VNADES (11.88 mg/g DW) and Ac (24.99 mg/g DW) from *E. prunastri* compared to the VNADES extract from *P. furfuracea* (over 35 mg/g DW of atranorin) suggests that a saturated solution was not reached in the VNADES extract from *E. prunastri*.

**Table 3:** Extraction efficiency of lichen metabolites from seven lichen species using VNADES (menthol/camphor, 1.5:1 molar ratio) and acetone as solvents under ultrasound-assisted extraction conditions (15 min, 40 °C, solvent-to-material ratio of 100:1).

Lichen species	Metabolites	Type of solvents				Significance
		VNADES		Acetone		
*H. physodes*	Physodalic acid	32.3	± 2.15	35.6	± 3.09	ns
	3-Hydroxyphysodic acid	35.0	± 6.26	46.3	± 1.88	**
	Physodic acid	30.0	± 1.35	35.9	± 1.63	**
	Atranorin	12.5	± 1.19	15.9	± 1.87	ns
	Chloroatranorin	3.1	± 0.39	4.9	± 1.04	*
*E. prunastri*	Evernic acid	29.3	± 7.54	47.7	± 0.90	*
	Atranorin	11.9	± 6.55	25.0	± 5.08	*
	Chloroatranorin	2.8	± 1.87	8.9	± 1.48	*
*C. uncialis*	Usnic acid	7.7	± 1.09	8.0	± 1.02	ns
*X. parietina*	Physcion	4.0	± 2.27	2.7	± 0.34	ns
	Atranorin	1.0	± 0.34	0.2	± 0.08	*
*P. adscendens*	Atranorin	11.7	± 2.62	29.0	± 3.74	**
*P. furfuracea*	Evernic acid	2.4	± 0.26	1.3	± 0.09	**
	Atranorin	35.6	± 4.06	56.8	± 10.06	*
	Chloroatranorin	0.233	± 0.0257	0.283	± 0.0331	ns
*P. sulcata*	Salazinic acid	28.74	± 3.249	34.74	± 8.969	ns
	Atranorin	2.32	± 0.437	5.29	± 2.582	ns

Data are mean mg/g DW ± SD (*n* = 3), ns—nonsignificant, *p < 0.05, **p < 0.01.

### Potential loss of metabolites in VNADES extracts: effects of evaporation and lyophilisation

One of the main limitations of using traditional NADES is their low vapor pressure, which makes it impossible to obtain a dry residue of extracts by evaporation of the solvent^33^. VNADES provide a solution to this problem, as the vapor pressure at 25 °C is 0.064 mm Hg and 0.65 mm Hg for menthol and camphor, respectively. This makes it possible to evaporate menthol and camphor mixtures (similarly to other VNADES consisting of essential oil components) e.g., by freeze-drying or in a rotary evaporator.

**Fig. 5:**
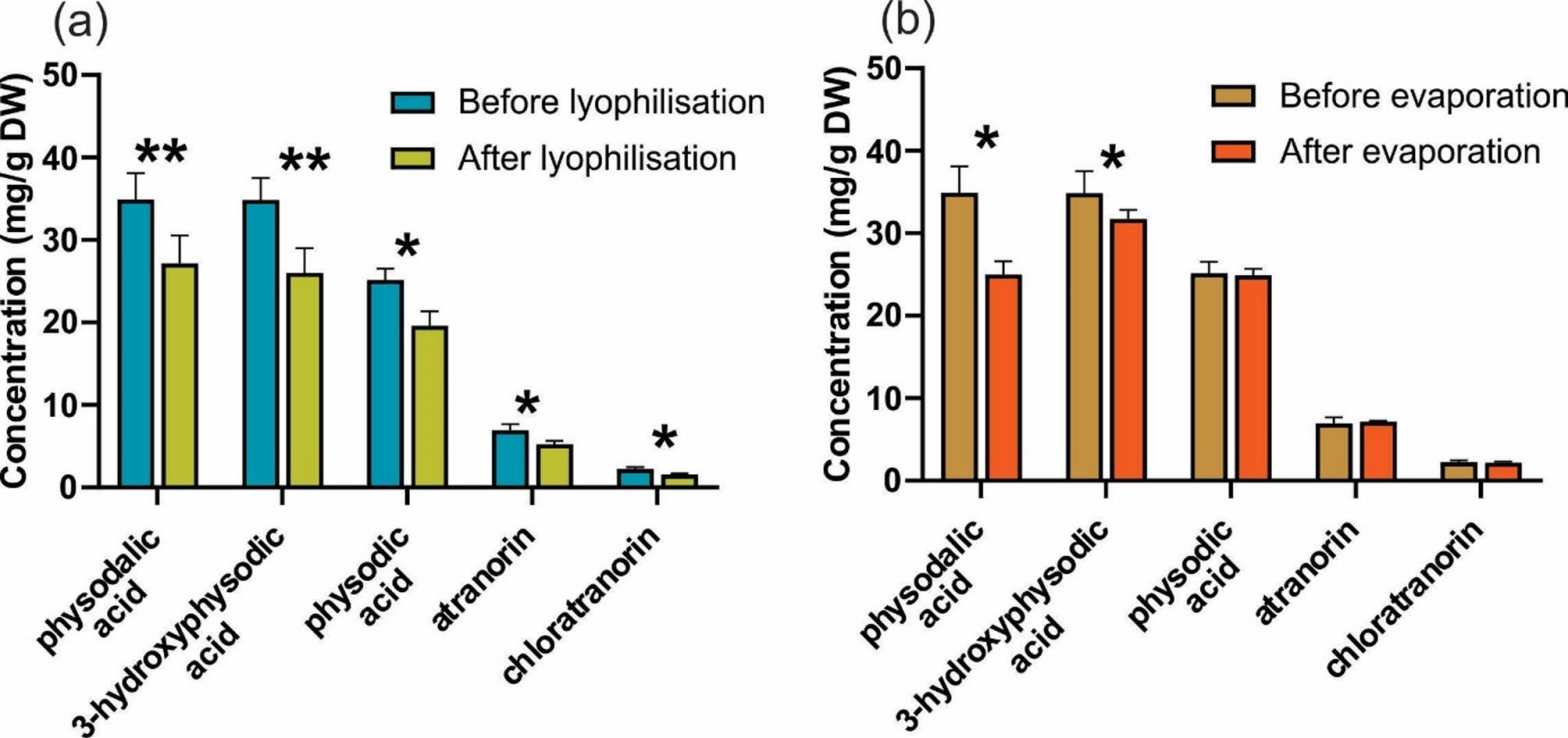
Influence of the extractant (menthol/camphor 1.5:1 molar ratio) evaporation technique on the content of lichen metabolites in *Hypogymia physodes* extracts. Panel (**a**) presents the loss of analytes as a result of the freeze-drying process of the extracts, panel (**b**) as a result of evaporation in a rotary evaporator.

In this study, extracts obtained from *H. physodes* using a menthol/camphor mixture (1.5:1 molar ratio) were evaporated, and then the contents of lichen acids in the dry residues were determined and compared with the initial contents. It was shown that lyophilisation caused losses in the content of all five compounds determined, and they were the highest in the case of physodalic acid and 3-hydroxyphysodic acid (Fig. 5a). In the case of these compounds, losses were also observed after evaporation of extracts in a rotary evaporator, however, this method did not cause the loss of physodic acid, anthranorin and chloratranorin (Fig. 5b). There is no literature data on the vapor pressure of the compounds determined, however, it can be assumed that physodalic acid and 3-hydroxyphysodic acid are characterised by higher volatility than the other three compounds. Lower losses of analytes in extracts evaporated in a rotary evaporator are probably related to the applied low vacuum parameters (30 mBar), in contrast to the lyophilisation process in which a vacuum of 0.01 mBar was obtained. Despite the noted losses in the analyte contents, the proposed methods allow for obtaining dry extract residues, and the use of a rotary evaporator enables a relatively quick and cost-effective alternative to lyophilisation for VNADES evaporation process.

## Materials and methods

### Chemicals and reference standards

HPLC-grade acetonitrile (ACN), trifluoroacetic acid (TFA ≥ 99.0%), Ac (≥ 99.9%), EtOAc (≥ 99.5%), atranorin (phyproof^®^), physcion (phyproof^®^), (+)-usnic acid (98%), DL-menthol (≥ 95%), and (±)-camphor (≥ 95.5%) were obtained from Sigma-Aldrich (St. Louis, MO, USA). Water was purified using by ULTRAPURE Milli-pore Direct-Q^®^3UV–R system (Merck Millipore, Billerica, Massachusetts, USA). Standards of salazinic and evernic acids were purchased from Cayman Chemical Company (Michigan, USA). 3-Hydroxyphysodic, physodalic, and physodic acids were isolated from *H. physodes* using the previously described method^21^. The content of chloroatranorin was indirectly estimated based on the calibration curve of atranorin.

### Plant material

**Fig. 6:**
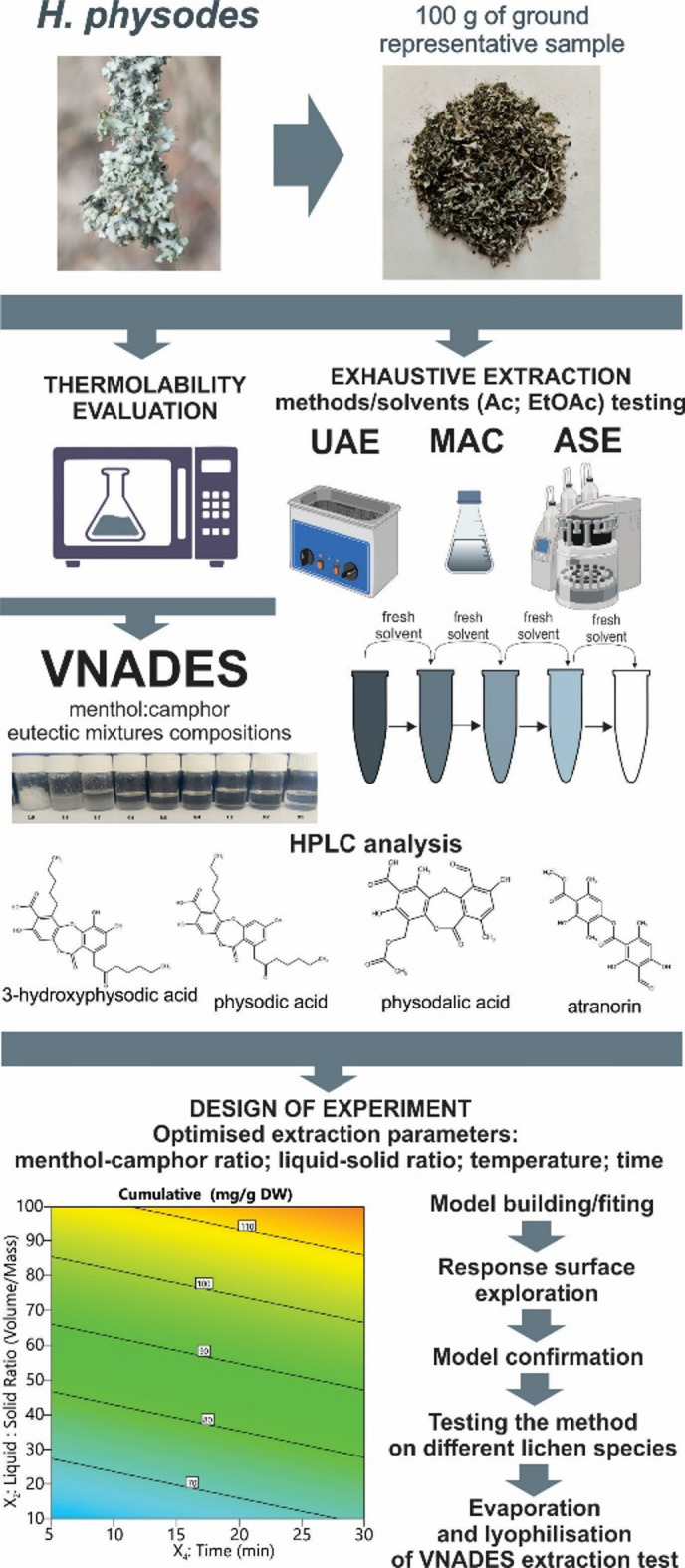
Overall research plan.

A total of seven species of lichens were identified, belonging to five distinct families: *Hypogymnia physodes* (L.) Nyl. (Parmeliaceae), *Evernia prunastri* (L.) Ach. (Usneaceae), *Cladonia uncialis* (L.) Weber ex F.H. Wigg. (Cladoniaceae), *Xanthoria parietina* (L.) Th. Fr. (Teloschistaceae), and *Physcia ascendens* (Fr.) H. Olivier. (Physciaceae), *Pseudevernia furfuracea* (L.) Zopf (Parmeliaceae), *Palmeria sulcata* Taylor (Parmeliaceae) were collected in Spring 2024 in Lublin Voivodeship, Poland (Suppl. Table 1). The species was verified by co-author lichenologist Dr. Hanna Wojciak. Although 7 species of lichens were collected, only *H. physodes* was used to evaluate the efficiency of extractants and extraction methods in metabolite extraction, as well as to optimise extraction using VNADES. The collected samples were manually cleaned and air-dried, after which the lichen samples were stored at 4 °C. Prior to extraction and analysis, the lichen thalli were powdered using a laboratory grinder IKA A11 (IKA-Werke, Staufen, Germany). The overall design of the research was given in Fig. 6.

### Preparation of VNADES

VNADES were obtained by mixed the menthol and camphor in the appropriate molar ratios, and heating in a water bath (60 min at 60 °C). VNADES were obtained using molar ratios of menthol to camphor in the range 1:9 to 9:1. The VNADES obtained are shown in Supplementary Fig. 2. In this study, the term “VNADES” is understood as referring to natural, volatile, low-melting mixtures. These systems are not yet sufficiently characterized from a physicochemical standpoint—in particular, the compositions at the eutectic point and their deviations from theoretically calculated values have not been specified.

### Thermolability assessment

The thermolability of lichen-derived compounds isolated from *H. physodes* was carried out by using two methods. The first method is based on the use of an Accelerated Solvent Extractor (ASE) (Thermo Scientific Dionex, ASE-350, New York, USA). The solution of lichen compounds was exposed to temperatures of 40 °C, 60 °C, and 80 °C, respectively at a pressure of approx. 1500 psi for 15 min in 22 mL Dionex stainless steel extraction cell. Due to the possibility of some of the solvent evaporating from the solvent bottle, the solution was analysed immediately before and after the procedure. The percentage of the compound remaining in the solution after the temperature treatment with respect to the initial solution (Eq. 1), was calculated according to the formula:

1 $${\text{CR\% ~}} = {\text{~}}\frac{{CT}}{{\frac{{CI1 + CI2}}{2}}} \times ~100\%$$

CR% - percentage concentration of analyte remaining after temperature exposed, relative to the initial amount; CT - concentration of the analyte in the solution exposed to temperature; CI1 - initial concentration of the analyte in the reservoir solution before analysis; CI2 - initial concentration of the analytic in the reservoir solution after analysis.

### Lichen metabolites extraction

All extractions (for each technique and extractant) were performed in triplicate. The extracts obtained were analysed by HPLC according to the methodology described in Sect. 3.6. To determine extraction yields, 10 mL of each extract was transferred to weighing dishes and evaporated under a nitrogen stream.

#### Ultrasound-assisted extraction (UAE)

A total of 0.25 g of *H. physodes* thallus was extracted with 25 mL of Ac or EtOAc using an ultrasonic bath (Sonorex RK 512 H, Bandelin, Berlin, Germany) at a frequency of 35 kHz and a temperature of 25 °C for 15 min. Temperature stability was ensured by a recirculating chiller (MultiTemp III, Pharmacia Biotech AB, Uppsala, Sweden). The samples were centrifuged, the supernatant was collected, supplemented to a total volume of 35 mL suitable solvent, and the plant material was extracted with a fresh portion of extractant. The extraction was repeated five times.

#### Maceration (MAC)

A total of 0.25 g of *H. physodes* thallus was poured with 25 mL of Ac or EtOAc. The sample was stirred and set aside in the dark at room temperature. After 24 h, the samples were centrifuged, the supernatant was collected, supplemented to a total volume of 35 mL suitable solvent. The plant material was then flooded with a fresh portion of extractant. Extraction was performed five times (5 × 24 h).

#### Accelerated solvent extraction (ASE)

The *H. physodes* thallus was extracted using an Accelerated Solvent Extractor (ASE; Thermo Scientific Dionex, ASE-350, New York, USA). A total of 0.25 g of ground lichen thallus was placed in a 22-mL Dionex stainless steel extraction cell. The extraction was carried out at a pressure of 1500 psi using Ac and EtOAc as solvents. The extraction temperature was set to 40 °C, with a static time of 15 min. The obtained extract was then supplemented to a total volume of 35 mL with the suitable solvent.

### Chromatographic analysis

A VWR Hitachi Chromaster 600 chromatograph with a PDA detector and EZChrom Elite software 3.2.0 (Merck, Darmstadt, Germany) was used for chromatohraphic analysis. The sample were analysed on an RP18e LiChrospher 100 column (25 cm × 4.0 mm i.d., 5 μm particle size) (Merck, Darmstadt, Germany), maintained at a temperature of 30 °C. The mobile phase consisted of water (solvent A) and ACN (solvent B), containing 0.025% TFA. The following gradient elution was applied: 0.0–30.0 min A 80%, B 20%; 30.1–60.0 min A 50%, B 50%; 60.1–70.0 min A 25%, B 75%; 70.1–79 min A 0%, B 100%; 79.1–85 min A 80%, B 20%. The flow rate was 1 mL/min and data were collected between 200 and 400 nm. The identity of compounds was established by comparing their retention times and UV spectra with corresponding standards. Quantitative analysis was performed at λ = 256 nm. Additionally, the metabolites were identified using Ultra-High Performance Liquid Chromatogram (UHPLC) 1290 Infinity Series II coupled with an Agilent 6224 electrospray ionization/time-of-flight mass detector (ESI/TOF) (Agilent Technologies, Santa Clara, CA, USA) according to the protocol described before^50^.

### Evaporation of extracts on VNADES base

#### Evaporation by freeze-drying

A 5 mL sample of the extracts was frozen at − 80 °C (Eppendorf CryoCube; Eppendorf AG, Hamburg, Germany) and freeze-dried at 0.01 mBar for 72 h (Alpha 2–4 LDplus Freeze Dryer; Martin Christ, Osterode am Harz, Germany). The dry residues were then weighed, dissolved in Ac, and analysed according to the method described in Sect. 3.6.

#### Evaporation in a rotary evaporator

5 mL of the sample was transferred to a round-bottom flask, 500 mL of deionized water was added and installed in a Hei-Vap Expert Control rotary evaporator (Heidolph Instruments GmbH & Co. KG, Schwabach, Germany). The evaporation process was carried out at 40 °C and 30 mBar for 3 h. The dry residues were dissolved in Ac and analysed according to the method described in Sect. 3.6.

### Optimization of extraction using VNADES using DoE and statistical analysis

The D-optimal design was used to investigate the impact of four independent numerical factors: *X*_*1*_: menthol/camphor molar ratio (1.5-9); *X*_*2*_: liquid/solid ratio (volume/mass) (10–100); *X*_*3*_: temperature of extraction (20–40 °C); *X*_*4*_: time of extraction (5–30 min). Polynomial models were constructed separately for all compounds. The models’ significance was evaluated using ANOVA. Furthermore, model adequacy was evaluated by calculating fit statistics such determination coefficient (R2), adjusted R2, predicted R2, and adequate precision. The finalised model was then utilised for numerical optimisation. Through a series of experiments, the models were confirmed with the predicted values (*n* = 3). The difference between factors were evaluated using one-way or two-way ANOVA followed by Tukey’s test at 0.05 probability level (*n* = 3). The statistical analysis was performed using Statistica ver. 13.3.0.3 (Tibco Software Inc., Palo Alto, CA, USA) while DoE procedure was carried out using Design Expert ver. 13 (Stat-Ease Inc. Minneapolis, MN, USA).

## Conclusions

The presented study showed the importance of optimising the extraction methods of bioactive compounds from *Hypogymnia physodes* and various lichen species, considering the complex structure of living organisms and low solubility of their metabolites. The combination of ASE with Ac was found to be the most effective method for obtaining high yields of lichen components, including physodalic and physodic acids, 3-hydroxyphysodic acid, atranorin, chloroatranorin. At the same time, long MAC with Ac also showed comparable effectiveness to Ac in ultrasonic extraction for the recovery of depsidones with the same ASE extraction efficiency of EtOAc. The use of VNADES as an alternative green extraction method was shown to be the most progressive approach for the extraction of lichen’s secondary metabolites, especially in increasing selectivity while reducing the dependence on traditional solvents. However, careful selection of optimal parameters such as the ratio of menthol and camphor (in this case), L/S ratio, extraction time and temperature should be considered, which affect the efficiency of extraction of bioactive compounds. Furthermore, the study highlights the importance of the choice of the extraction drying method, which is important for further pharmacological tests to develop standardisation parameters. In this case, the use of a rotary evaporator was identified as a rapid and cost-effective alternative to lyophilisation for the evaporation of VNADES, providing a practical solution for obtaining dry extracts with reduced compound losses. According to our data, such an assessment of the effect of the drying method on the chemical profile of components in lichens is presented for the first time. Given the findings, further developments in VNADES-based extraction should improve the efficiency of compound extraction and selectivity. In addition, this approach can optimise resource use and reduce waste. The antimicrobial and cytotoxic potential of individual compounds and the total extracts of *H. physodes* and species highlights the potential of ongoing research for pharmaceutical developments.

## Electronic supplementary material

Below is the link to the electronic supplementary material.

Supplementary Material 1

## Data Availability

The datasets used and/or analysed during the current study are available from the corresponding author upon reasonable request. For data access inquiries, please contact S. Dresler at: slawomir.dresler@umlub.pl.
