# Supplementary material for: Extraction of lichen bioactive compounds using volatile natural deep eutectic solvents and comparative analytical approaches
Source: Sci Rep. 2025 Jul 2;15:22742. doi: 10.1038/s41598-025-08069-0 (PMC12215910; doi:10.1038/s41598-025-08069-0)
Supplement: Supplementary file 1 — Supplementary Material 1 [file 41598_2025_8069_MOESM1_ESM.docx]

**Supplementary Materials**

**Extraction of lichen bioactive compounds using volatile natural deep eutectic solvents and comparative analytical approaches**

Dresler S.^1,2*^, Baczewska I.^1^, Mykhailenko O.^3,4,5^, Zidorn Ch.^5,6^, Sowa I.^1,^ Wójciak M.^1^, Feldo M.^7^, Wójciak H.^8^, Hanaka A.^2^, Strzemski M.^1*^

^1^ Department of Analytical Chemistry, Medical University of Lublin, Chodźki 4a, 20-093 Lublin, Poland

^2^ Department of Plant Physiology and Biophysics, Institute of Biological Sciences, Maria Curie-Skłodowska University, Akademicka 19, 20-033 Lublin, Poland

^3^ Department of Pharmaceutical Chemistry, National University of Pharmacy, 61168 Kharkiv, Ukraine; o.mykhailenko@nuph.edu.ua

^4^ School of Pharmacy, University College London, 29-39 Brunswick Square, London WC1N 1AX, UK

^5^ Department of Pharmaceutical Biology, Kiel University, 24118 Kiel, Germany

^6^ Division of Pharmaceutical Biotechnology, Department of Pharmaceutical, Biology and Biotechnology, Wroclaw Medical University, Borowska 211,

50-556 Wrocław, Poland.

^7^ Department of Vascular Surgery, Medical University of Lublin, Staszica 11, 20-081 Lublin, Poland

^8^ Institute of Biological Sciences, Maria Curie-Skłodowska University, Akademicka 19, 20-033 Lublin, Poland

* Corresponding authors:

e-mail: slawomir.dresler@poczta.umcs.lublin.pl; maciej.strzemski@umlub.pl


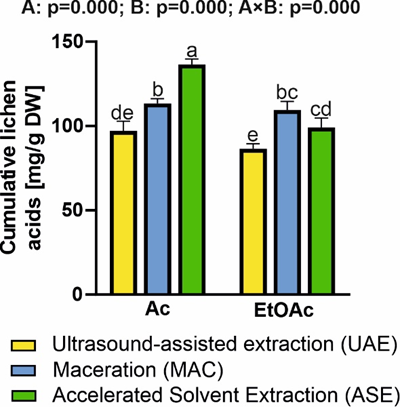


**Suppl. Figure 1.** Effect of solvent type (acetone – Ac; ethyl acetate – EtOAc) and extraction method (ASE – Accelerated Solvent Extraction; MAC – Maceration; UAE – Ultrasound-Assisted Extraction) on the cumulative lichen acids content from five extraction steps. Columns marked with different lowercase letters represent significant differences based on Tukey's post-hoc test (p < 0.05). Two-way ANOVA results: p-values indicate the effects of factors: A (extraction method), B (solvent type), and A x B (interaction between method and solvent). Data are presented as means (n=3) ± standard deviation (SD).

**
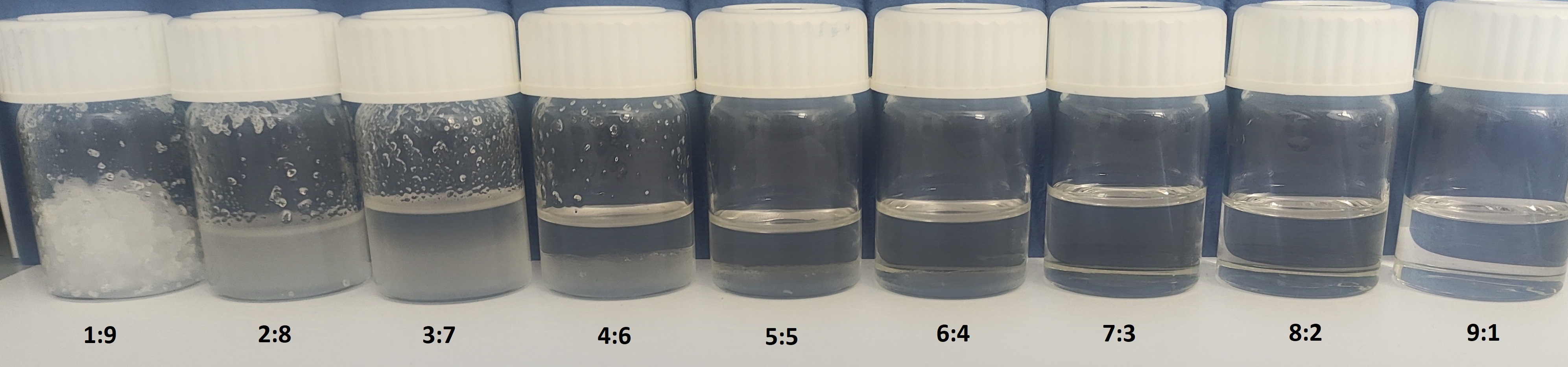
Suppl. Figure 2.** Appearance of prepared menthol:camphor eutectic mixtures in a molar ratio from 1:9 to 9:1.

**Suppl. Table 1.** Details of sampling areas.

| Species | Localization | Coordinates DMS |
| --- | --- | --- |
| *Hypogymnia physodes* | Janowskie Forest | N 50°39’49,90”  E 22°21’07.10” |
| *Evernia prunastri* | Mircze | N 50°39’39.67”  E 23°52’22.83” |
| *Cladonia uncialis* | Sobibór Forest | N 51°27’34.20”  E 23°36’32.90” |
| *Xanthoria parietina* | Mircze | N 50°39’39.67”  E 23°52’22.83” |
| *Physcia ascendens* | Mircze | N 50°39’39.67”  E 23°52’22.83” |
| *Pseudevernia furfuracea* | Janowskie Forest | N 50°39’49,90”  E 22°21’07.10” |
| *Palmeria sulcata* | Mircze | N 50°39’39.67”  E 23°52’22.83” |
|  |  |  |
